# Supplementary figures and images for: Endothelin receptor Aa regulates proliferation and differentiation of Erb-dependent pigment progenitors in zebrafish
Source: PLoS Genet. 2019 Feb 27;15(2):e1007941. doi: 10.1371/journal.pgen.1007941 (PMC6392274; doi:10.1371/journal.pgen.1007941)

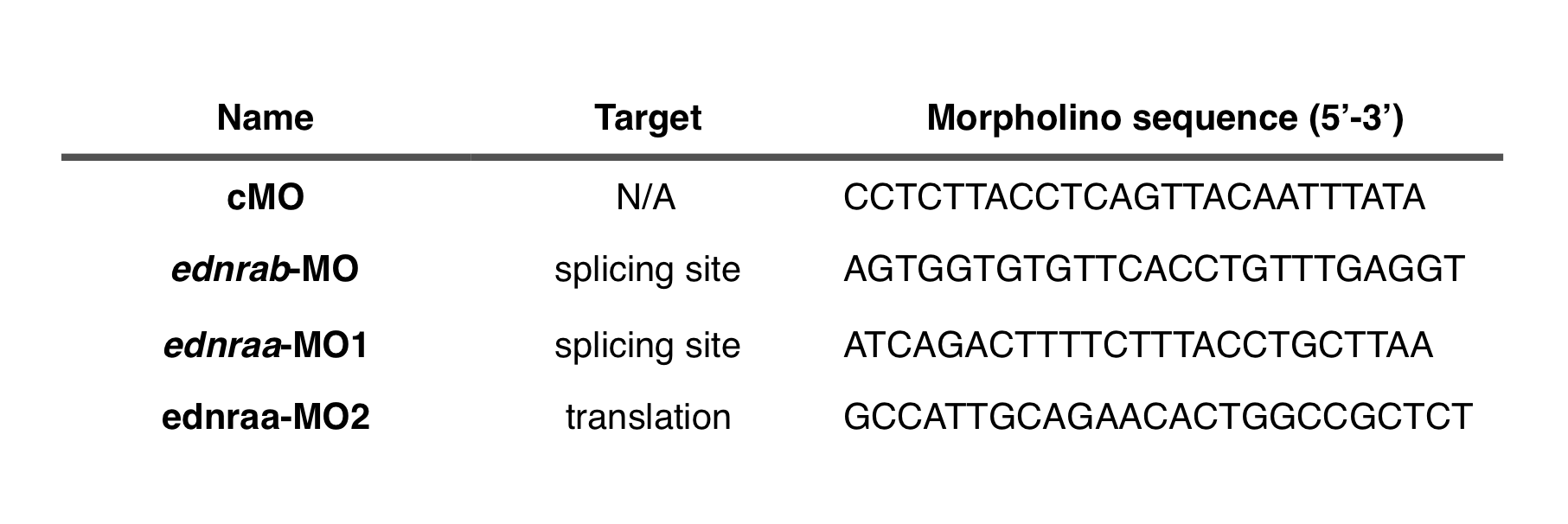

Supplement: S1 Table — A random sequence morpholino (cMO) provided by Gen Tools and a splice-blocking morpholino against ednrab (ednrab-MO) were used as control. ednraa was targeted with either a splice-blocking morpholino (ednraa -MO1) or a translation-blocking ednraa morpholino (ednraa-MO2). Scale bar = 100 μm (A-C). (TIFF) [file pgen.1007941.s001.tiff]

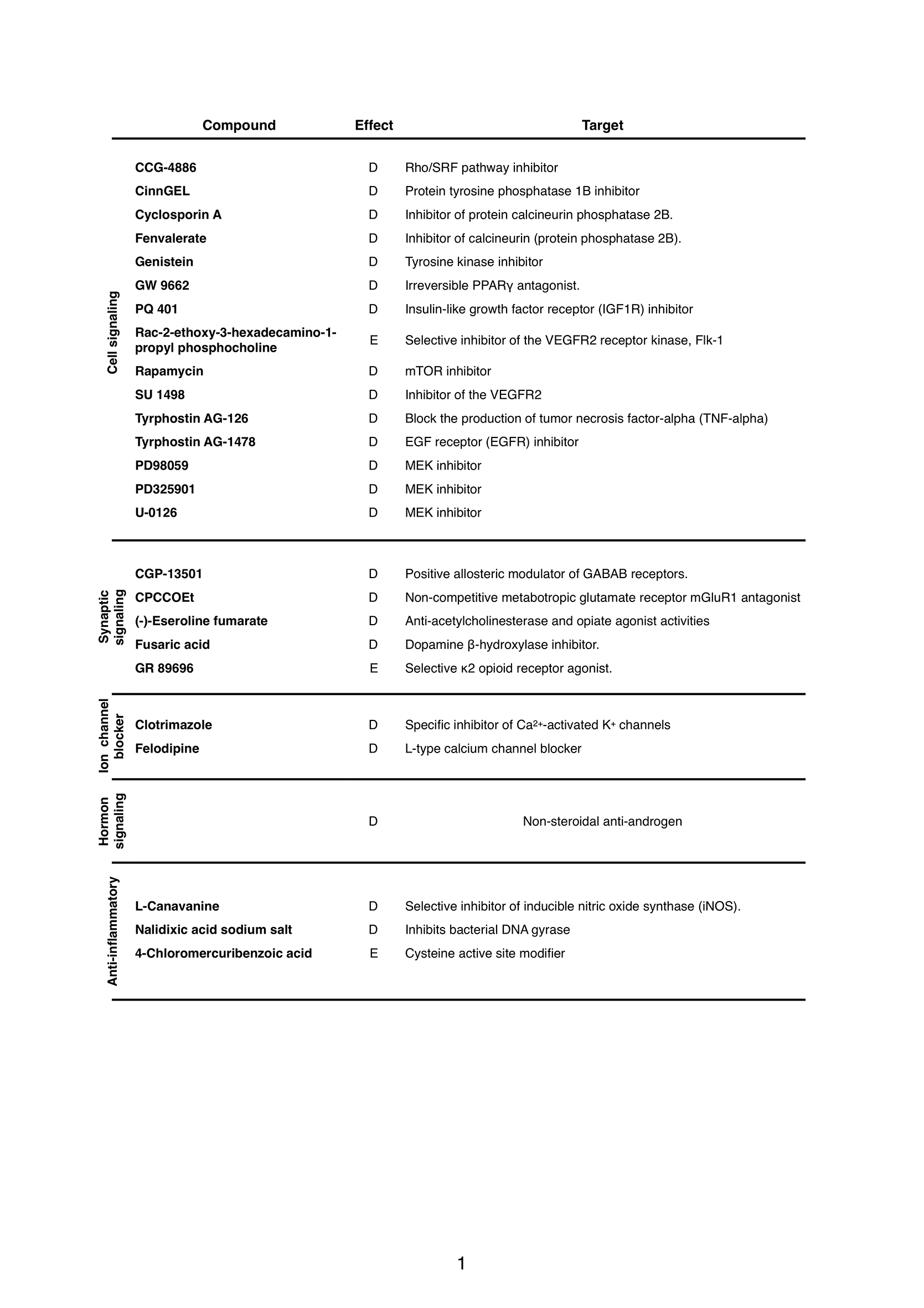

Supplement: S2 Table — Name and known targets of small molecules that enhance (E) or rescue (R) the pde phenotype. (TIFF) [file pgen.1007941.s002.tiff]

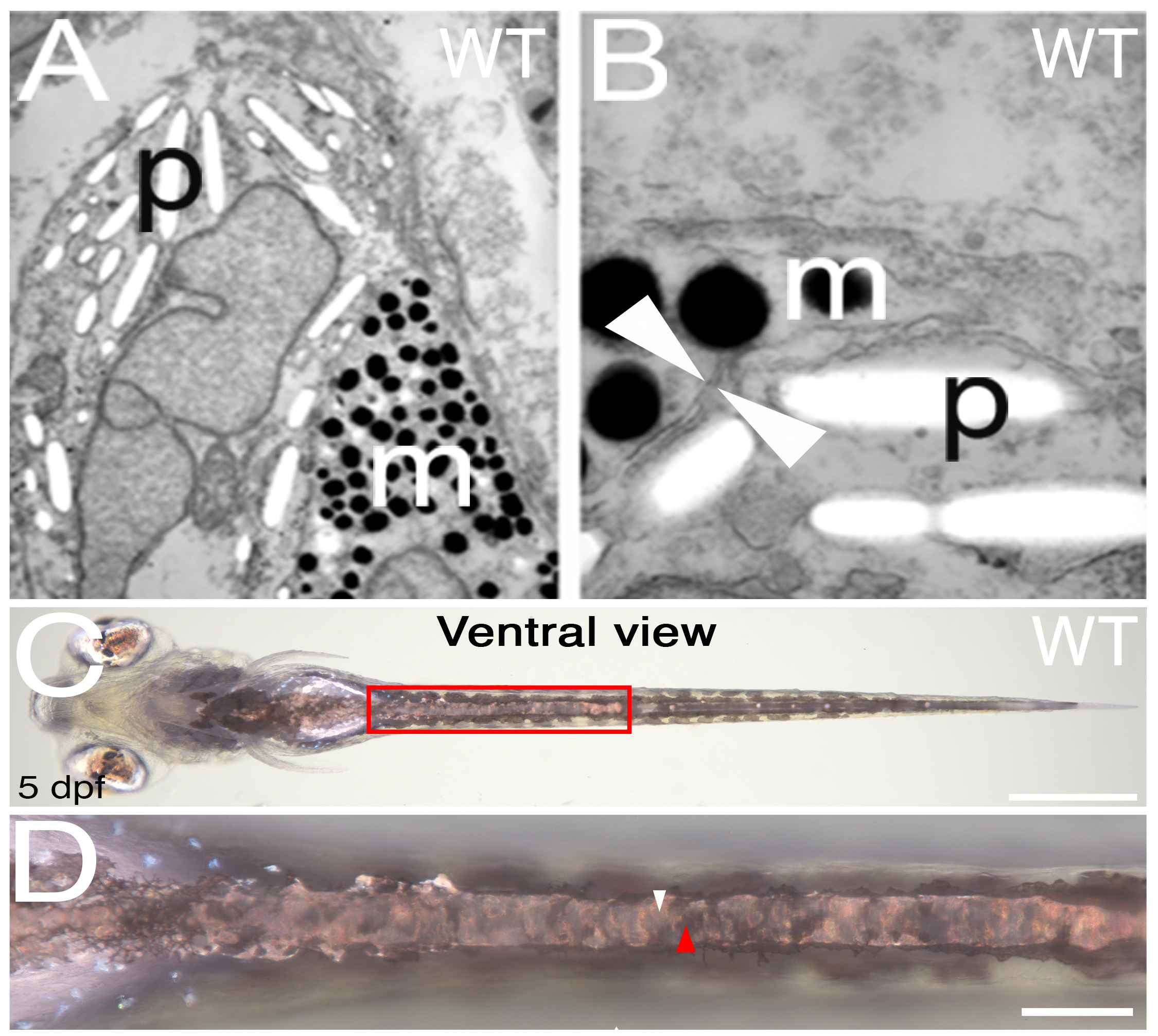

Supplement: S1 Fig — Transmission electron photomicrographs of melanocytes and iridophores in the WT yolk sac stripe ectopic pigment cells in pde mutants. A and B show two examples of melanosomes (m) and reflecting. platelets (p) separated by a double membrane (white arrowheads). Bright-field image of ventral view (C) and close up of the area in the red box (D) of WT fish, shows yolk sac stripe. Continuous layer of iridophores is indicated by white arrowhead in D, closely associated black melanocytes forming contiguous layer immediately dorsal to iridophores is indicatted by red arrowhead. Scale bars = 500 μm (C) and 100 μm (D). (TIF) [file pgen.1007941.s003.tif]

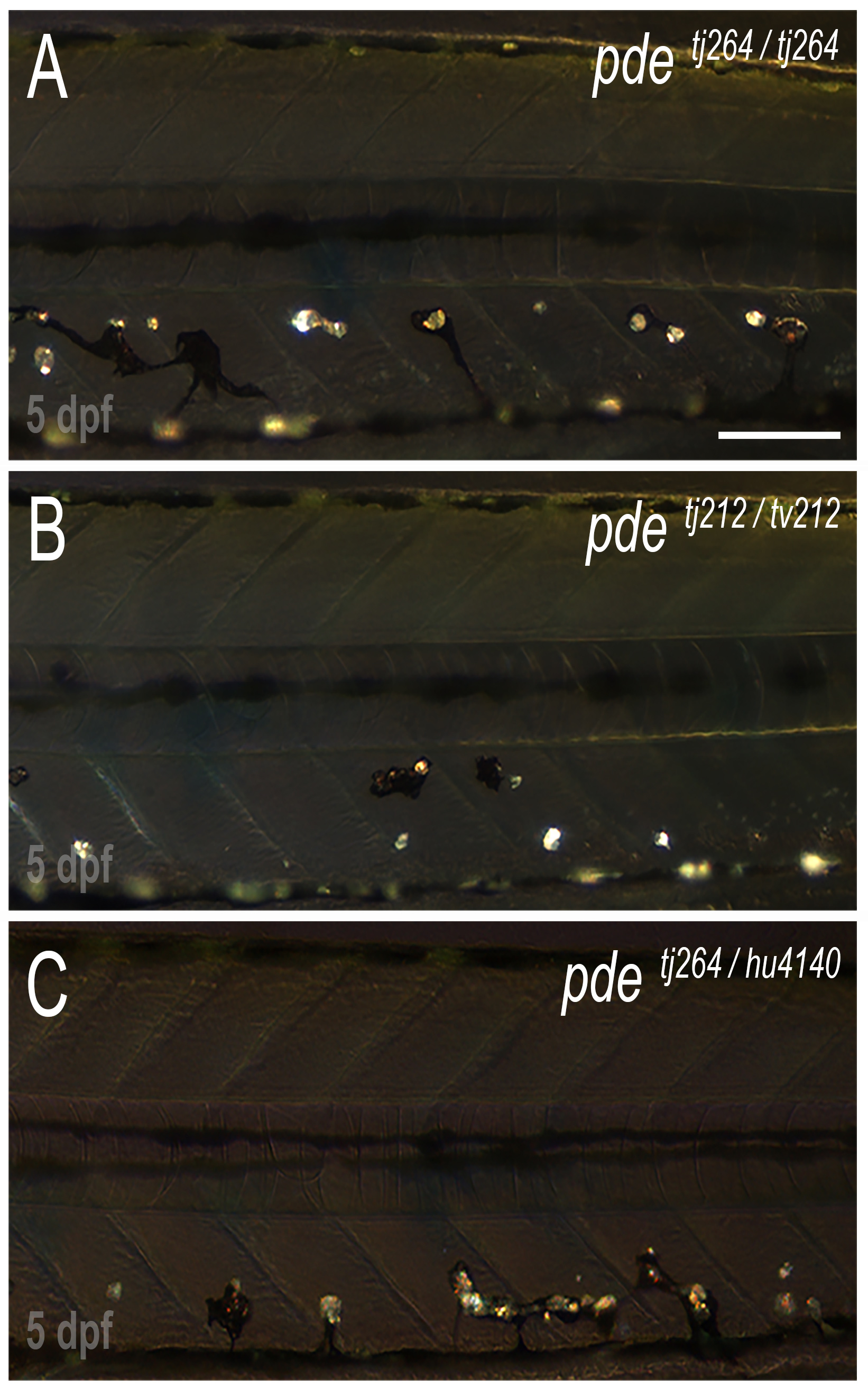

Supplement: S2 Fig — Overview of early larval pigment phenotype at 5 dpf of pdetj262/tj262 (A), pdetj262/tv212 (B) and pdetj262/hu4140(C). All three allele combinations show ectopic melanocyte and iridophores in the ventral medial pathway of the posterior trunk. (TIF) [file pgen.1007941.s004.tif]

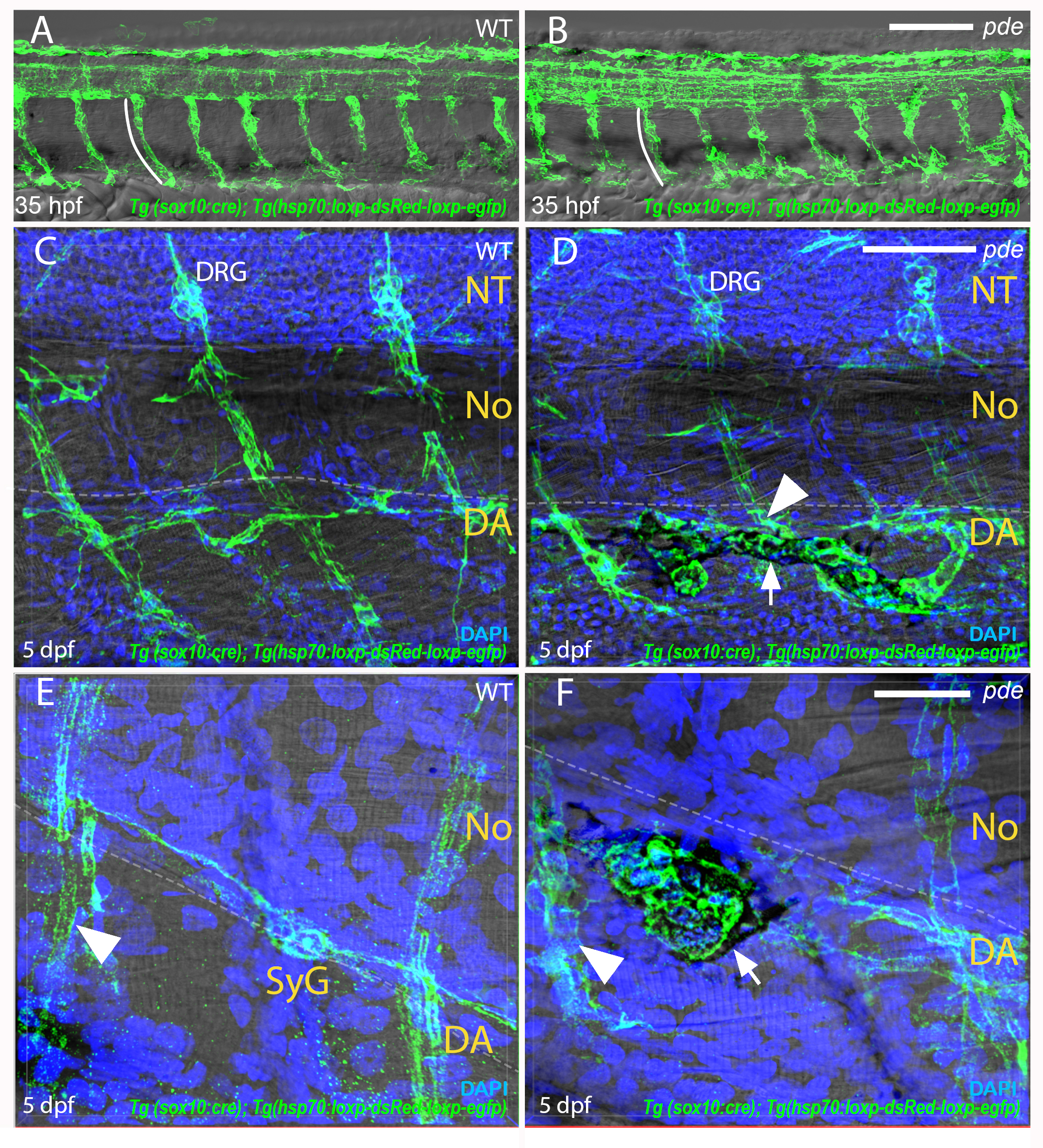

Supplement: S3 Fig — Labelling of neural crest derivatives with GFP using the transgenic line Tg(-4725sox10:cre)ba74; Tg(hsp:loxp-dsRed-loxp-LYN-EGFP) shows no difference between 35 hpf WT fish (A) and pde mutants (B), neural crest cells migrate ventrally in a intersegmental arrangement (white line in A and B). 5 dpf pde mutant larvae show ectopic pigment cells (white arrow in D) associated with the spinal nerve projections (arrowheads in D) that emerge from the dorsal root ganglia (DRG). Ectopic pigment cells (white arrows) are also associated with the sympathetic ganglion (SyG) chain that forms perpendicular to the spinal nerve projections (white arrowhead in E and F) and ventral to the notochord (No). Guided by DIC image, dorsal edge of the dorsal aorta (DA) is highlighted with a dashed white line in C-F. Neural tube (NT). DAPI labels nuclei (blue). Scale bar = 25 μm (A and B), 50 μm (C and D) and 15 μm (E-F). (TIF) [file pgen.1007941.s005.tif]

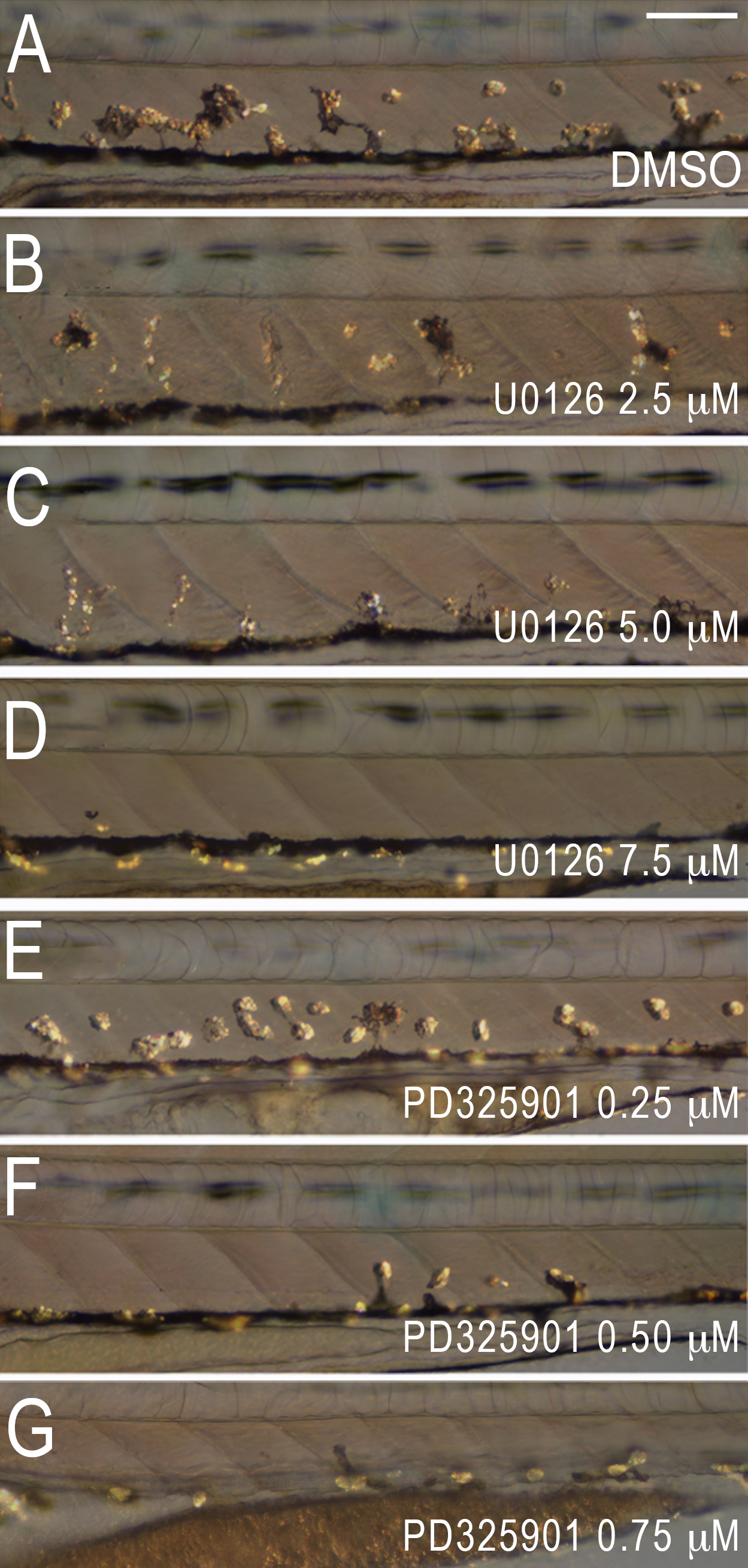

Supplement: S4 Fig — Treatment with increasing concentrations of the MEK inhibitors U0126 (2.5–7.6 μM) and PD 325901 (0.25–0.75μM), from 6–96 hpf, shows increasing rescue of the ectopic pigment cells. Scale bar = 100 μm (A-G). (JPG) [file pgen.1007941.s006.jpg]

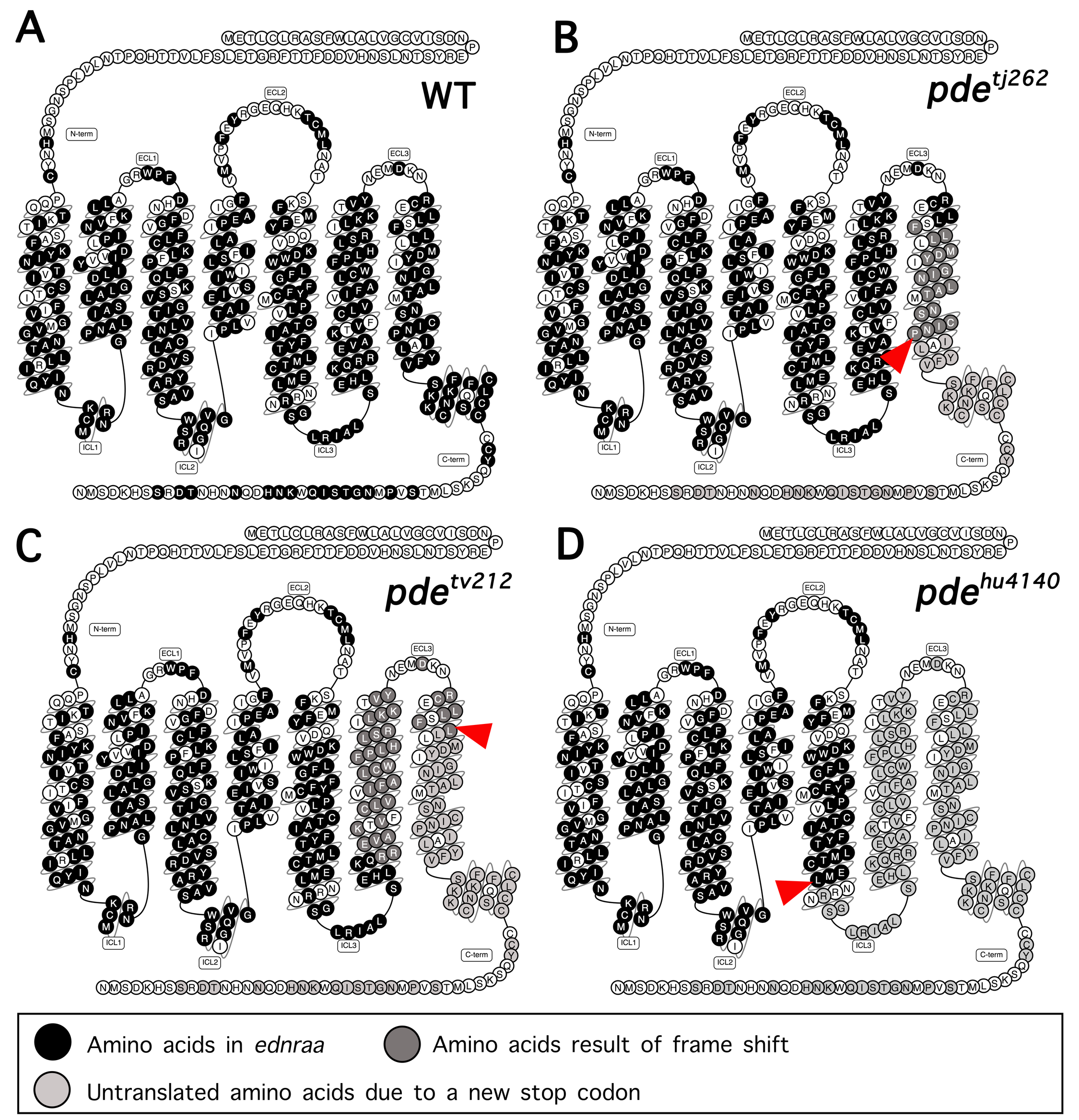

Supplement: S5 Fig — Scheme shows 2D structure of the human ETA receptor, with identical amino acids of the zebrafish EdnrAa receptor shown in black for the WT allele (A), pdetj262 (B), pdetv212 (C) and pdehu4140(D). In the mutant alleles, changed coding regions are indicated as follows: Amino acids changed due to a shift in the reading frame are shown in dark grey and amino acids that are absent since lie after new stop codon (red arrowheads) are shown in light grey. (TIF) [file pgen.1007941.s007.tif]

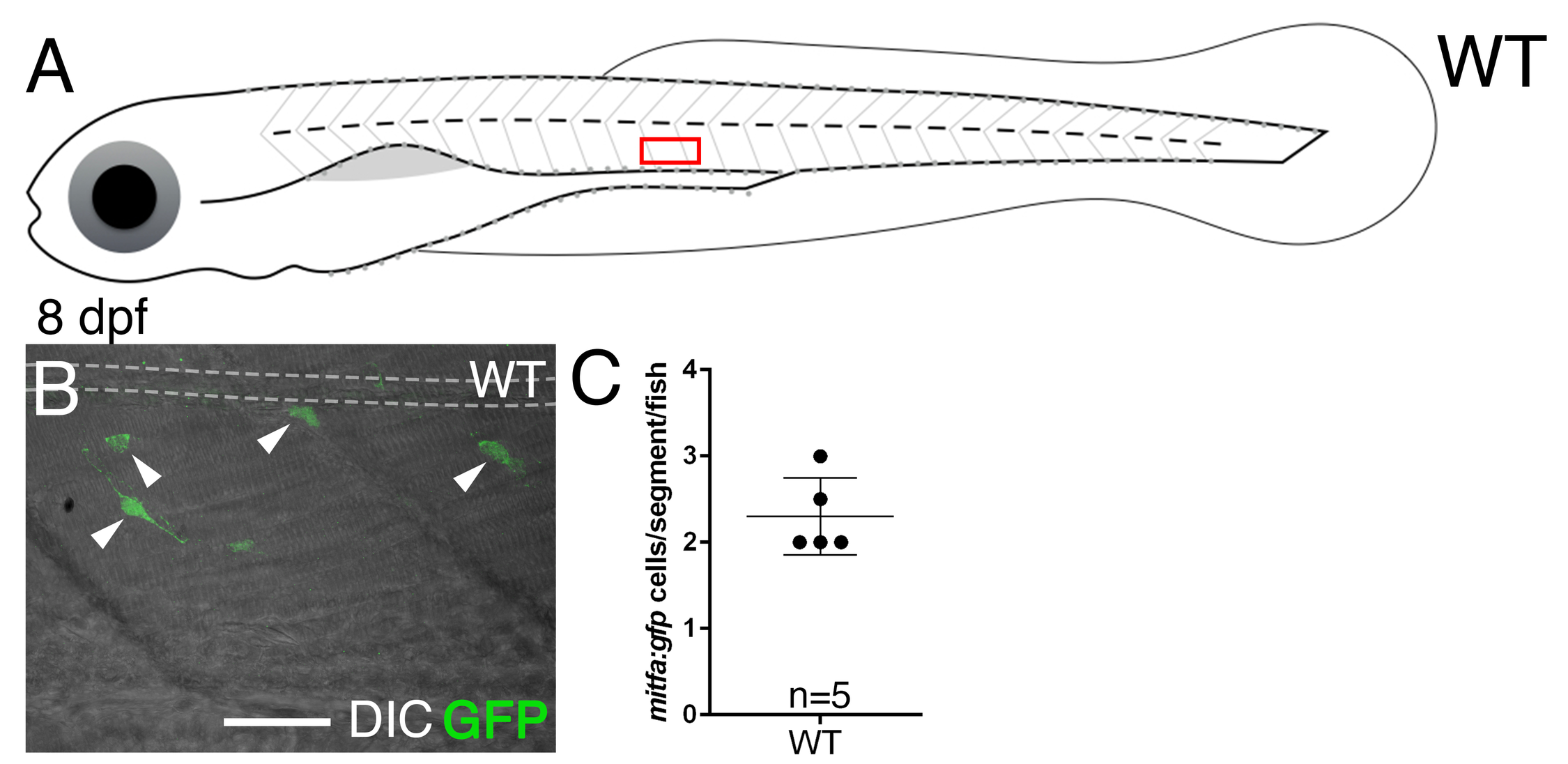

Supplement: S6 Fig — (A) Scheme shows 8 dpf fish, with the red box indicating the area where mitfa:gfp positive cells in the ventral trunk were found. (B) GFP+ cells are readily found in the vicinity of the dorsal aorta throughout the posterior trunk and anterior tail at 8 dpf; superimposed DIC image shows these cells are not melanised. (C) Quantitation of GFP+ cells from a random posterior trunk segment in each of 5 fish, given as mean±s.d. = 2.3±0.44 (n = 5). (TIF) [file pgen.1007941.s008.tif]
